# Predicting the Need for Transition from Pediatric to Adult Pain Services: A Retrospective, Longitudinal Study Using the Electronic Persistent Pain Outcome Collaboration (ePPOC) Databases

**DOI:** 10.3390/children10020357

**Published:** 2023-02-10

**Authors:** Joel Champion, Matthew Crawford, Tiina Jaaniste

**Affiliations:** 1 Department of Pain, Sydney Children’s Hospital, Randwick, NSW 2031, Australia; 2 School of Clinical Medicine, University of New South Wales, Kensington, NSW 2052, Australia

**Keywords:** chronic pain, youth, adult, outcome, treatment responder, transition

## Abstract

A proportion of youth with chronic pain do not respond to interdisciplinary pain management and may require transition to adult pain services. This study sought to characterize a cohort of patients referred to pediatric pain services who subsequently required referral to an adult pain service. We compared this transition group with pediatric patients eligible by age to transition but who did not transition to adult services. We sought to identify factors predicting the need to transition to adult pain services. This retrospective study utilized linkage data from the adult electronic Persistent Pain Outcomes Collaboration (ePPOC) and the pediatric (PaedePPOC) data repositories. The transition group experienced significantly higher pain intensity and disability, lower quality of life, and higher health care utilization relative to the comparison group. Parents of the transition group reported greater distress, catastrophizing, and helplessness relative to parents in the comparison group. Three factors significantly predicted transition: compensation status (OR = 4.21 (1.185–15)), daily anti-inflammatory medication use (OR = 2 (1.028–3.9)), and older age at referral (OR = 1.6 (1.3–2.17)). This study demonstrated that patients referred to pediatric pain services who subsequently need transition to adult services are a uniquely disabled and vulnerable group beyond comparative peers. Clinical applications for transition-specific care are discussed.

## 1. Introduction

Chronic pain in children and adolescents is known to predict poorer outcomes in adulthood across multiple health and socioeconomic measures [1,2,3,4,5,6,7,8,9]. Whilst significant gains have been made in the understanding of chronic pain, there is a knowledge gap for patients experiencing persistent pain and disability despite intervention, especially when this occurs during the consequential transition period from adolescence to young adulthood [10,11,12]. There is increasing recognition of the need for effective transition-specific services to improve the health and socioeconomic outcomes for young people with chronic pain [13].

Interdisciplinary pain management has been found to be effective for pediatric chronic pain conditions, whether delivered as intensive inpatient [14,15,16] or outpatient [17,18] programs. Intensive interdisciplinary pain management approaches have been found to result in a range of pain-related functional, psychosocial, and economic benefits [15,16,19]. There is evidence that these benefits last at least 12 months for 56% to 88% of pediatric patients [16,19,20,21]. This means that there remains a large proportion of patients who are non-responders, and there is a paucity of research focused on this group. Hechler and colleagues found that more than 30% of pediatric patients did not achieve long-term improvement following intensive interdisciplinary interventions [22]. Another study identified 22% of youth who did not respond to an intensive multimodal pain intervention [23].

Many services lack the resources to implement intensive inpatient pediatric chronic pain programs, with interdisciplinary outpatient programs being more common [24]. In a sample of children with chronic pain receiving multimodal outpatient interventions, pain intensity, pain-related disability, and use of unhelpful coping strategies were significantly reduced at 3 months [17]. Moreover, almost 70% of children were able to attend school regularly at the 12-month follow-up [17]. However, 12% remained in treatment at 12 months, requiring ongoing interventions.

There is emerging research to identify patient characteristics associated with various outcome profiles following interdisciplinary pediatric pain management. Hirschfeld et al. [23] found that following an intensive multimodal pain intervention, pediatric patients who achieved short-term improvement at 3 months, but not at 12 months, had higher levels of anxiety and depression at baseline [23]. Banez et al. found that patients with larger reductions in pain intensity scores at 2–4-year follow-up after a pediatric intensive interdisciplinary pain intervention had higher initial pain scores and shorter chronicity (<12 months versus >12 months) relative to those with smaller reductions in pain scores [25]. Of the variables assessed, Banez et al. did not find any factors that significantly predicted non-response to treatment [25]. Simons et al. [19] found that non-response for pain intensity following an intensive pain rehabilitation program was predicted by higher initial levels of pain intensity, older age, higher anxiety, and lower patient readiness to change [19]. However, there were no significant predictors of functional disability. Other studies recommend further investigation to explain variance in treatment response, with potential factors including pre-treatment pain characteristics, gender, and emotional variables [22], as well as patient and carer pain cognitions and behaviors [26,27,28].

For youth who do not respond to available pain interventions, potentially requiring transition to adult pain services, the personal and societal consequences are considerable [29]. Ongoing chronic pain through adolescence and into early adulthood may have an impact on normal adolescent development, including emerging autonomy, maturation of peer relations and sexuality, and educational and vocational functioning [12]. The risk for these vulnerable youth is that these factors compound in adolescence and cement a trajectory of disadvantage into adulthood [9,12]. Thus, it would be valuable to identify which pediatric patients are at risk of ongoing pain, subsequently requiring transition to adult pain services. Those engaged with the health system at this age also need to acquire a range of self-management skills to transition from family-oriented pediatric health care to individually focused adult health services [30]. The acquisition of these skills may be complicated by low self-efficacy, helplessness, catastrophizing, anxiety, and depression, which are commonly associated with chronic pain [6,9,12,31].

An emerging body of research has focused on the potential value of transition-specific care for youth with chronic conditions [32,33,34]. These programs strive to help youth with the challenges of managing their health care during a unique developmental period, whilst also navigating the changes from a pediatric to adult health care system [13,35]. Further work is needed to develop and evaluate transition-specific programs for chronic pain patients [12,36]. If transition-specific care can offer the chance to mitigate a trajectory of disadvantage for adolescents with chronic pain, it is important to gain an understanding of early risk factors predicting the need for referral to adult pain services [7]. To our knowledge, there are no quantitative studies that utilize linkage data between pediatric and adult pain services to analyze patients who require adult pain services following engagement with pediatric pain services. A clearer knowledge of risk factors may enable the development of clinical screening tools to inform timely and targeted intervention for those most likely to experience ongoing pain and disability into adulthood.

The aims of this study were three-fold. First, we sought to provide much needed demographic, clinical, and psychosocial data on pediatric patients with chronic pain who go on to require transition from pediatric to adult pain services. Second, we aimed to compare this transition cohort, at the time of initial pediatric pain clinic assessment, with peers in pediatric services. Third, we sought to identify patient and carer factors at the time of assessment at a pediatric pain service that predict the subsequent need for transition to an adult pain service. It was hypothesized that the transition group, relative to comparative peers in pain services, would report more severe pain intensity and related disability, greater pain chronicity, more comorbidities, greater medication usage, and higher levels of carer distress, and that these factors are identifiable at referral and predict the need for transition to adult pain services.

## 2. Materials and Methods

### 2.1. Study Design

This was a retrospective study utilizing a de-identified key, linking prospectively collected data derived from the pediatric and adult electronic Persistent Pain Outcomes Collaboration (ePPOC) databases. Release of the data was approved by the ePPOC Data Access Working Group. Written consent from participants was not required as data analysis and linkages were prepared by ePPOC personnel employed by Wollongong University and identifiable data were not released to researchers.

The study was approved by the Sydney Children’s Hospital Network Human Research Ethics Committee (2021/ETH11168, 17 August 2021).

### 2.2. Cohort

Data were extracted from the Pediatric electronic Persistent Pain Outcomes Collaboration (PaedePPOC) [37,38], established in 2014 and administered by the Australian Health Services Research Institute (AHSRI) and the University of Wollongong. Pediatric pain services across Australia contribute de-identified patient and carer data to the PaedePPOC database. The adult electronic Persistent Pain Outcomes Collaboration (ePPOC) data were used to select which pediatric patients subsequently transitioned to an adult pain service. Use of a linkage key enabled this to be performed whilst preserving patient anonymity [37]. The PaedePPOC database captures data from pediatric pain services in Australia which are almost entirely provided in public health institutions. The ePPOC database captures the majority of adult pain services; however, a large fraction of adult care is provided with private services, some of which are outside the ePPOC database.

All new patients who attended a PaedePPOC participating pediatric pain service between 2014 and 2020 and were eligible to transition to adult services were included in this study. Those younger than 16 years of age throughout the study period were excluded as they were not eligible to transition to an adult pain service. The pediatric pain transition cohort consisted of eligible patients identified by linkage key to have subsequently registered at an adult pain service. The comparator group consisted of eligible peers in pediatric pain services who did not require subsequent referral to adult pain services.

### 2.3. Data Extraction

The following data were obtained from the PaedePPOC database initial assessment questionnaires for all pediatric participants eligible for transfer to adult services: age at initial assessment, gender, country of birth, duration of pain, pain description, primary pain site, source of pain, compensation status (presence vs. absence of a legal compensation case), comorbidities and disabilities, health service utilization, and daily analgesic use. Data from the following standardized questionnaires were also retrieved.

Modified Brief Pain Inventory (MBPI). The MBPI is a four-item self-report and carer proxy-report questionnaire assessment tool designed to evaluate pain intensity, rating the worst, least, and average pain intensity over the past week, as well as current pain intensity on a numeric rating scale from zero being “no pain” to ten being “the worst pain you can imagine” [39]. Pain intensity has been classified in the PaedePPOC database as mild (0–4), moderate (5–6), or severe (7–10) [37].

Functional Disability Inventory (FDI). The FDI is a 15-item self-report questionnaire designed to assess function and disability in children with chronic pain. Items are rated with regard to how difficult respondents found each activity in the past 2 weeks, with response options ranging from “no trouble” (scored 0) to “impossible” (scored 4). The FDI is a valid and reliable assessment of pain-related disability in children and adolescents [24,40,41,42].

Pediatric Quality of Life Inventory (PedsQL). The PedsQL is a 23-item measure of health-related quality of life in children and adolescents, consisting of 4 sub-scales: physical (8 items), emotional (5 items), social (5 items), and school (5 items) functioning. A 5-point response scale is used to indicate how often each item has been a problem over the past month, from “never” (scored 0) to “almost always” (scored 4). The PedsQL is a valid and reliable measure of health-related quality of life for use with pediatric samples [43,44]. If more than 50% of items in the scale were missing, the scale scores were not calculated. “At risk status” for impaired HRQOL for child self-report is regarded as a total score below 69.7 and below 65.4 for adult proxy-report [44].

Bath Adolescent Pain Parent Impact Questionnaire (BAP-PIQ). The BAP-PIQ is a 61-item parent-report measure which assesses the psychosocial impact of caring for a child with chronic pain on the life of a parent/carer. The BAP-PIQ has eight sub-scales measuring parental depression, anxiety, child pain-related catastrophizing, self-blame and helplessness, partner relationship, leisure functioning, parental behavior, and parental strain. A higher score in each domain indicates greater impairment [45]. The BAP-PIQ is a reliable and valid assessment of the psychosocial impacts of parenting an adolescent with chronic pain [45,46].

### 2.4. Statistical Analyses

Statistical analysis software (SAS v9.4) was used for data analysis. Comparative demographic, clinical characteristics, health care utilization, medications use, and questionnaire scores were examined for the transition and comparison groups. Continuous variables were presented as means (*M*) with standard deviations (*SD*), age with medians and interquartile ranges (*IQR*), whilst categorical variables were presented as number (*n*) and percentages (%). Data were inspected for deviation from normal distribution with Q-Q plots and histograms. Data with normal distribution were analyzed with two-sample *t*-tests, and non-parametric data with the Wilcoxon rank sum test. Pearson chi-square tests were used to assess group differences for categorical variables. The level of significance was set with a *p* value of 0.05.

Variables that were found to be significantly different were checked for multi-collinearity and entered into a logistic regression model as possible predictors for the need for transition.

## 3. Results

### 3.1. Patient Characteristics

Patient characteristics for the transition group (*n* = 86) and comparison groups (*n* = 1541) are shown in Table 1. The transition group was older but otherwise similar, with the majority being female and born in Australia/New Zealand.

The pain condition experienced by the transition group was more frequently related to a compensation case (*n* = 6; 9.5%) relative to the comparison group (*n* = 29; 2.5%, *p* = 0.001). The transition group had a non-significant trend towards more frequent mental health conditions (*p* = 0.08) and pain “always being present” (*p* = 0.055).

### 3.2. Pain Clinical Characteristics and Responses

Health service utilization data did not conform to a normal distribution on examination of Q-Q plots and histograms, whilst all other variables including pain intensity, pain relating functioning, medication use, parental impact, and case involving compensation were normally distributed.

Health care utilization (HCU) in the preceding three months was significantly higher for the transition group relative to the comparison group for family doctor appointments, allied health appointments, emergency department visits, and diagnostic tests (see Table 2).

Daily anti-inflammatory use was significantly greater in the transition cohort (*n* = 26, 41.9%) relative to the comparison group (*n* = 262, 24.0%, *p* = 0.002), but not for anti-neuropathic or opioid analgesics (see Table 3).

Patient self-reported scores of pain intensity and functioning are shown in Table 4. As shown in Table 4, the transition group reported significantly higher pain severity (*p* = 0.02), higher worst pain scores (*p* = 0.004), worse functional disability (*p* = 0.03), and lower quality of life in all functional domains (all *p*’s < 0.05) relative to the comparison group.

Table 4 also shows carer reported data for patient pain intensity, disability, functioning, and parental impact. Although responses for the transition group were poorer on all measures relative to the comparison group, not all reached statistical significance. Scores were significantly worse for the transition cohort across all domains of functioning (PedsQL), excluding the physical domain, relative to the comparison group.

Carers in the transition group reported significantly higher pain-related catastrophizing (*p* < 0.01) and helplessness (*p* ≤ 0.01) relative to the comparison cohort.

Variables shown to be significantly different between the two cohorts on analysis of means or distribution were assessed with bivariate Pearson correlations to check for multi-collinearity (see Supplementary Table S1).

### 3.3. Logistic Regression Analysis

Results from the regression modelling, as presented in Table 5, revealed that the significant risk factors for the subsequent need to transition were older age at initial pain clinic referral, compensation status, and daily use of anti-inflammatory medications. Patients involved with compensation were 4.2 times as likely to transition compared to those for whom there was no compensation. However, compensation cases in pediatric pain services were infrequent in both groups, and the small numbers lead to a much wider 95% confidence interval. Patients taking anti-inflammatory medication daily were twice as likely to transition compared to those who used anti-inflammatory medications less frequently or not at all. For the remaining variables, the odds ratios indicated that these variables were not statistically significant at a 0.05 level of significance.

FDI and Peds QL had a correlation coefficient greater than 0.7 (see Supplementary Table S1), suggesting the potential presence of multi-collinearity. However, refitting the model with each of these factors removed in turn provided very similar results, suggesting that multi-collinearity was not significant.

Results of the Hosmer–Lemeshow test, *p*-value = 0.356, indicate there was no evidence of a lack of fit between the model and the data. The event rates were similar to that expected and observed in sub-groups, indicating a well-calibrated model. The C-statistic for area under the curve (AUC) was 0.779, indicating that the model provides an acceptable level of discrimination between those who transitioned and those who did not.

## 4. Discussion

This study provides new data to enhance understanding about the cohort of youth attending pediatric chronic pain services who subsequently go on to transition to adult pain services. Both the transition and comparison groups reported moderate pain severity, severe worst pain, and in the “at risk” range [44] for self-reported and carer-reported quality of life. However, in line with the first aim of the study and consistent with the hypothesis, the transition cohort was characterized as having significantly worse pain intensity and functional disability, more frequent health care utilization, and broad impairment across all measured functional domains relative to the comparison group. Contrary to the hypothesis, these were not significant statistical predictors for the need to transition. Nevertheless, univariate analyses highlight that pediatric patients with poorer pain and functioning at assessment are at greatest risk of ongoing pain problems requiring transition to an adult pain service. These findings are consistent with the results of Simon et al., who also found that elevated pain intensity prior to a pain intervention was a predictor of poor response [19].

The current study found that carer-reported maladaptive cognitions, such as pain-related catastrophizing and helplessness, were significantly higher in the transition cohort in univariate analyses, but not predictive of the need to transition in regression analysis. There is substantive research on the reciprocal effects of negative parent/carer and child dyad coping styles [47,48]. The literature suggests that carers’ cognitions and affective functioning may be modifiable risk factors for persistent pain through adolescence into adulthood. For example, parental catastrophizing has been found to be associated with the child’s level of pain-related disability independent of pain intensity [49,50].

Consistent with the existing research [19,29], it was found that older age at the time of referral predicted a need to transition to adult pain services. It is possible that this reflects reduced cognitive flexibility or readiness to change, and the ability to engage with a self-management treatment approach [19,51]. We also present previously unrecognized risk factors for transition, namely medicolegal compensation status and the daily use of anti-inflammatory medications. Pediatric chronic pain patients who were involved with a compensation case were over four times more likely to transition to adult pain services. Similarly, the process of seeking financial compensation in adults has been found to be related to worse pain scores and disability, and a poor prognostic factor for chronicity, outcomes following rehabilitation treatment programs, and return to work across a range of pain-related conditions [52,53,54]. Reasons for this are also likely to be relevant to pediatric patients and include stress relating to the adversarial nature of litigation, perceived injustice, and number of medical assessments. Although compensation cases are relatively uncommon among pediatric chronic pain patients [54], they should be considered as a risk for subsequent transition to adult pain services.

Daily anti-inflammatory medication use was associated with twice the risk of a need to transition. There is a lack of evidence for NSAID’s efficacy in children with chronic pain [5,55,56]. Continued NSAID prescription may reflect a familiarity with NSAID prescription relative to other pharmacological intervention for chronic pain in children amongst general medical practitioners, who were visited significantly more frequently by the transition cohort relative to the comparison group.

Health care utilization was high in both the transition and comparison groups, consistent with the existing studies of children with chronic pain [7,57,58,59,60]. The elevated rates of emergency department presentation and use of diagnostic services in the transition group are particularly notable, given these are not effective components of chronic pain management and may be suggestive of high levels of pain severity and pain-related distress [22]. From a health economic perspective, patients who continue to require extensive health care utilization into adulthood become a disproportionately costly group [60,61].

We present the first use of linkage data to study the referral characteristics of a pediatric population enrolled in pain services over the transition period to adult pain services. We have shown that patients referred to pediatric pain services who subsequently need transition to adult pain services are a uniquely vulnerable group. They experience worse pain intensity, distress, and impairment with disability across many aspects of their lives that is above and beyond that experienced by peers in pediatric pain services who are not referred to adult services. This study identified three referral characteristics, namely compensation status, daily anti-inflammatory use, and older age at referral, predictive of need for transition. Moreover, the study contributes to the paucity of quantitative research that risk stratifies children and adolescents in pain services who may need to transition to adult services.

### 4.1. Limitations

The results of this study should be considered in light of some limitations. The data captured only those patients who transitioned to ePPOC participating pain services and not all adult pain services. The transition group was on average one year older than the comparison group at the time of referral to a pediatric pain service. However, normative data for Australian children and adolescents referred to specialist pain centers do not show worse pain severity or related disability, across the 8–12-year and 12–18-year groups [62]. In addition, age was not highly correlated with pain severity or disability, meaning these variables are independent and the stepwise logistic regression enables assessment of effects above and beyond any due to age. We concluded that although the transition group was older, the findings of higher pain severity, pain-related disability, and worse quality of life than peers in pediatric pain services was independent of their age at the time of referral.

Within the database used, some populations may have been under-represented in pain clinics, due to difficulties in engaging in the programs offered. These may include youth with severe mental illness, severe intellectual disability, or not proficient in the English language. In addition, there is inequity of service provision for pediatric patients with chronic pain in Australia, with those living in the most socioeconomically disadvantaged areas being referred the least [63]. The results from the current study, therefore, may not be generalizable to these under-represented populations.

The current study was not able to analyze the role of resilience factors, such as self-esteem, family cohesion, social connectedness [64], optimism [65,66], or psychological flexibility [67], which may modify treatment outcomes. Moreover, the risk-resilience model recognizes potentially modifiable resilience factors, such as mindfulness [66], non-judgmental focus and acceptance [68], readiness to change [19], and self-regulation [66], that can mitigate the consequences of persistent pain.

### 4.2. Strengths

The key strengths of the current study were that it utilized a large, multi-center data set, it was not compromised by research volunteer bias (given that questionnaire completion was a requirement of pain clinic assessment), and it used a control group. Some inherent weaknesses common to many retrospective cohort studies, including recall bias and inconsistencies in data collection, were reduced by ePPOC data being prospectively collected, using standardized assessment tools. The use of ePPOC linkage data also mitigated loss to follow-up. This is important because there are few studies that investigate predictive factors for treatment failure in pediatric chronic pain and all suffer from poor retention rates [19,25,29,69]. The study utilized a longitudinal study design, allowing for the analysis of predictive factors of subsequent outcomes.

### 4.3. Clinical Implications

Implementing transition-specific care for adolescents with persistent pain is in its infancy. This study has characterized a cohort of pediatric patients needing transition to adult pain services so that future research goals may elucidate obstacles and facilitators to successful transition. The current findings suggest that patients can be risk stratified for need to transition at referral to pediatric pain services, by screening for cases involving compensation, daily use of NSAIDs, and older age at referral. By identifying patients who are at risk of poorer pain trajectories, and longer recovery times, there is a need for clinical researchers to determine whether targeted interventions may be directed at this cohort, with the aim of improving their outcome trajectory. Consideration should be given to whether transition care should start earlier to better accommodate the needs of these youth.

High parental catastrophizing and helplessness are prevalent amongst carers for adolescents who are subsequently referred to adult pain services. High carer catastrophizing and helplessness have been shown to partially mediate both protective parental behaviors and child-reported pain intensity [70]. We speculate that these carers may be less able to support their child in the use of effective pain coping strategies. Interventions targeting maladaptive caregiver cognitions may be beneficial for adolescents, as demonstrated for other childhood epochs [70,71,72]. The current study lends support to the importance of screening for parental catastrophizing and helplessness as potential therapeutic targets.

## 5. Conclusions

Children and adolescents referred to pediatric pain services who subsequently need transition to adult pain services are a uniquely vulnerable and disabled group. At initial referral to pediatric pain services, they experience significantly higher pain intensity and disability, have higher health care utilization, lower quality of life, and higher carer distress, beyond that experienced by patients and carers in pediatric pain services who do not need referral to adult services.

We have identified three baseline characteristics that predict the need for referral to adult pain services, namely compensation status, daily use of NSAIDs, and older age at the time of pediatric referral. This research highlights the potential for pediatric pain services screening for children more likely to need transition to adult pain services, and to facilitate the development of early and specific transition care for those that do.

## Figures and Tables

**Table 1:** Characteristics of patients in the transition cohort relative to the comparison group.

Variable	Transition Cohort	Comparison Group	*p*-Value
	(*n* = 86)	(*n* = 1541)	
Age			<0.001
Mean (SD)	15.4 (1.3)	14.4 (1.6)	
Median	16	15	
IQR	15–16	13–15	
Range	11–18	9–19	
Sex, *n* (%)			0.210
Male	17 (19.8)	397 (25.8)	
Female	69 (80.2)	1144 (74.2)	
Country of birth, *n* (%)			0.380
Australia/New Zealand	62 (95.4)	1074 (92.4)	
Other	3 (4.6)	88 (7.6)	
Site of main pain, *n* (%)			0.730
Back	11 (19.0)	217 (20.6)	
Head	11 (19.0)	179 (17.0)	
Abdomen	11 (19.0)	163 (15.5)	
Arms	6 (10.3)	69 (6.5)	
Knees	5 (8.6)	93 (8.8)	
Other	14 (24.1)	333 (31.6)	
Duration of pain, *n* (%)			0.970
Less than 3 months	5 (7.9)	98 (8.8)	
3–12 months	18 (28.6)	318 (28.6)	
More than 12 months	40 (63.5)	699 (62.7)	
Pain description, *n* (%)			0.055
Always present	44 (84.6)	730 (72.5)	
Other	8 (15.4)	277 (27.5)	
Source of pain, *n* (%)			0.780
injury	29 (46.0)	481 (43.5)	
after surgery	23 (36.5)	452 (40.9)	
other	11 (17.5)	173 (15.6)	
Episode related to compensation, *n* (%)			0.001
Yes	6 (9.5)	29 (2.5)	
No	57 (90.5)	1114 (97.5)	
Chronic disease, *n* (%)			0.130
Yes	20 (23.3)	260 (16.9)	
No	66 (76.7)	1281 (83.1)	
Mental health condition, *n* (%)			0.080
Yes	27 (31.4)	356 (23.1)	
No	59 (68.6)	1185 (76.9)	
Cancer *			>0.050
Yes	<5 (<5.8)	25 (1.6)	
No	>81 (>94.2)	1516 (98.4)	
Disability: Sight impairment, *n* (%)			0.950
Yes	5 (5.8)	92 (6.0)	
No	81 (94.2)	1449 (94.0)	
Disability: Hearing impairment, *n* (%) *			>0.050
Yes	<5 (<5.8)	18 (1.2)	
No	>81 (>94.2)	1523 (98.8)	
Disability: Intellectual impairment, *n* (%) *			>0.050
Yes	<5 (<5.8)	45 (2.9)	
No	>81 (>94.2)	1496 (97.1)	
Disability: Physical impairment, *n* (%)			0.250
Yes	9 (10.5)	110 (7.1)	
No	77 (89.5)	1431 (92.9)	

* denotes instances where cells have been confidentialized due to small cell sizes.

**Table 2:** Health care utilization of the transition cohort and comparison group.

Health Care Utilization, Mean (SD)	Transition Cohort	Comparison Group	*p*-Value *
General Practitioner	5.1 (4.4)	3.5 (4.3)	<0.001
Medical specialist	3.8 (5.7)	2.8 (3.6)	0.059
Allied health	5.8 (7.3)	3.9 (5.4)	0.011
Other therapists	1.5 (2.7)	1.3 (3.5)	0.156
Emergency department	1.9 (2.7)	1.0 (1.9)	0.011
Hospital admission	0.8 (1.6)	0.4 (1.2)	0.061
Diagnostic tests	2.7 (2.3)	2.1 (2.3)	0.017

* Wilcoxon two-sample test, two-sided test *p*-value.

**Table 3:** Medication use of the transition cohort and comparison group.

Medication, *n* (%)	Transition Cohort	Comparison Group	*p*-Value
Anti-inflammatory			0.002
Daily	26 (41.9)	262 (24.0)	
Other	36 (58.1)	831 (76.0)	
Medication for nerve pain			0.069
Daily	26 (44.8)	352 (33.2)	
Other	32 (55.2)	707 (66.8)	
Opioids (with or without codeine)			0.132
Daily	10 (16.4)	110 (10.3)	
Other	51 (83.6)	960 (89.7)	

**Table 4:** Carer and patient reported pain assessment questionnaire scores at referral.

	Transition Cohort Mean (SD)	Comparison Group Mean (SD)	*p*-Value
**Patient Reported Questionnaires**			
Brief Pain Inventory:			
Pain Severity	6.1 (1.6)	5.5 (1.9)	0.02
Worst Pain	8.2 (1.4)	7.7 (1.8)	0.004
Functional Disability Index (FDI)	29.7 (13.1)	26.4 (11.8)	0.03
PedsQL:			
Physical	33.4 (21.7)	39.3 (21.1)	0.03
Emotional	41.9 (23.1)	49.7 (22.9)	0.006
Social	55.6 (23.8)	65.2 (22.9)	<0.001
School	37.4 (25.3)	44.7 (22.3)	0.01
Psychological	44.9 (19.6)	53.2 (18.3)	<0.001
Total	40.9 (19.1)	48.4 (17.3)	<0.001
**Carer Reported Questionnaires**			
Brief Pain Inventory:			
Pain Severity	6.0 (1.7)	5.6 (1.8)	0.07
Worst Pain	7.8 (1.7)	7.6 (1.9)	0.4
Functional Disability Index (FDI)	29.7 (13.1)	26.4 (11.8)	0.03
PedsQL:			
Physical	32.3 (21.2)	37.0 (21.0)	0.09
Emotional	38.0 (20.7)	45.6 (21.0)	0.006
Social	54.6 (20.4)	60.5 (21.7)	0.04
School	34.4 (24.8)	43.0 (21.4)	0.003
Psychological	42.3 (17.8)	49.7 (16.8)	<0.001
Total	38.8 (17.5)	45.2 (16.2)	0.003
Bath Parent Impact Questionnaire:			
Depression	15.6 (6.4)	14.5 (6.4)	0.17
Anxiety	10.1 (4.9)	9.3 (5.1)	0.25
Catastrophizing	11.7 (4.2)	9.9 (4.3)	0.002
Helplessness	14.7 (5.5)	12.6 (6.3)	0.01
Partner relationship	10.9 (5.4)	10.7 (5.7)	0.79
Leisure functioning	17.1 (5.3)	16.4 (5.5)	0.33
Parental behavior	28.3 (5.0)	27.4 (6.0)	0.26
Parental strain	9.7 (5.8)	8.6 (5.1)	0.08
Total	115.2 (24.1)	108.1 (30.8)	0.06

**Table 5:** Logistic regression analysis predicting transition to an adult pain service.

Variable	Odds Ratio (OR)	95% Confidence Interval
Age	1.683	1.301–2.176
BPI Pain Severity	1.105	0.906–1.348
FDI Total	0.984	0.942–1.027
PedsQL Total	0.988	0.959–1.018
BAPPIQ Catastrophizing	1.063	0.961–1.175
BAPPIQ Helplessness	1.013	0.947–1.083
Anti-inflammatory medication		
Daily use	2.004	1.028–3.907
Other	1	
Compensation case		
Yes	4.218	1.185–15.008
No	1	

Hosmer–Lemeshow test, *p*-value = 0.3565. C-statistic (AUC) = 0.779.

## Data Availability

The data presented in this study are available upon reasonable request from the corresponding author.

## Supplementary Materials

The following supporting information can be downloaded at: https://www.mdpi.com/article/10.3390/children10020357/s1, Table S1: Bivariate correlations.
