# Supplementary material for: Predicting the Need for Transition from Pediatric to Adult Pain Services: A Retrospective, Longitudinal Study Using the Electronic Persistent Pain Outcome Collaboration (ePPOC) Databases
Source: Children (Basel). 2023 Feb 10;10(2):357. doi: 10.3390/children10020357 (PMC9955863; doi:10.3390/children10020357)
Supplement: Supplementary file 1 [file children-2182545-supplementary.pdf › children-2182545-supplementary.pdf]

Supplementary Materials

Variables shown to be significantly different between the two cohorts on analysis of means or distribution were assessed with bivariate Pearson correlations to check for multi-collinearity. Correlation coefficients greater than .7 were investigated for possible multi-collinearity. The variables found to be significantly different between groups were assessed as possible predictors of the need to transition to adult services using a logistic regression model. The predictors included in the model were: age, BPI pain severity, FDI total, PedsQL total, BAP-PIQ helplessness and catastrophizing, daily anti-inflammatory (NSAID) use, compensation case and health service utilization.

Table S1. Bivariate correlations.

	**Age**	**BPI Pain Severity**	**FDI**	**PedsQL Total**	**BAPPIQ Catastrophising**	**BAPPIQ Helplessness**	**HSU GP**	**HSU Hospital admissions**	**HSU Allied Health**	**HSU Emergency Department**
Age	1	0.018	0.061	-0.124	0.061	0.052	0.063	-0.047	0.068	-0.074
BPI Pain Severity		1	0.510	-0.402	0.154	0.226	0.205	0.004	0.113	0.097
FDI			1	-0.759	0.237	0.254	0.200	0.116	0.133	0.137
PedsQLTotal				1	-0.333	-0.344	-0.199	-0.122	-0.156	-0.126
BAPPIQ Catastrophising					1	0.661	0.144	0.118	0.021	0.141
BAPPIQ Helplessness						1	0.136	0.091	0.016	0.079
HSU GP							1	0.196	0.170	0.340
HSU Hospital admissions								1	0.106	0.565
HSU Allied Health									1	0.151
HSU Emergency Department										1
